# The prevalence of post-traumatic stress disorder in college students by continents and national income during the COVID-19 pandemic: a meta-analysis

**DOI:** 10.3389/fpsyg.2023.1129782

**Published:** 2023-05-12

**Authors:** Biao Hu, Xiling Yang, Xiaoqian Tuo

**Affiliations:** ^1^School of Marxism, Xi’an Jiaotong University, Xi’an, Shaanxi, China; ^2^Department of Obstetrics and Gynecology, The First Affiliated Hospital of Xi’an Jiaotong University, Xi’an, Shaanxi, China; ^3^Department of Gynecology, Shaanxi Provincial People's Hospital, Xi'an, Shaanxi, China

**Keywords:** PTSD, college students, mental health, COVID-19, meta-analysis

## Abstract

**Introduction:**

The present study aimed to provide a more accurate representation of post-traumatic stress disorder (PTSD) in college students during COVID-19 by performing meta-analyses by continents, national income, and study majors, and comparing the results with estimated pooled prevalence.

**Methods:**

Based on the guideline of PRISMA, literature was searched in PubMed, Web of Science, and Embase. The prevalence of PTSD was estimated through a random model based on the different continents and levels of national income, as well as study majors, and compared with the pooled prevalence of PTSD among college students.

**Results:**

Totally 381 articles were retrieved from electronic databases and 38 articles were included in the present meta-analysis. The results showed that the pooled prevalence of college students’ PTSD was 25% (95% CI: 21–28%). Prevalence estimates of PTSD among college students were statistically significant (*p* < 0.00001) when stratified with geographical regions, income levels, and study majors. In comparison with the pooled prevalence of PTSD (25%), subgroups of Africa and Europe, lower-middle-income countries, and medical college students possessed higher prevalence estimates.

**Discussion:**

The findings of the study showed that the prevalence of PTSD in college students worldwide during COVID-19 was relatively high and varied in different continents and countries with different income levels. Therefore, healthcare providers should pay attention to the psychologically healthy condition of college students during COVID-19.

## 1. Introduction

Around 16 years after the severe acute respiratory syndrome (SARS), another pathogenic coronavirus, the coronavirus disease-2019 (COVID-19) emerged in Wuhan City (Hubei Province, China) in 2019. Due to the serious situation, the World Health Organization (WHO) declared COVID-19 a global pandemic on March 11, 2020 (Si et al., 2021). Since the declaration of the outbreak, many countries adopted strict policies to control the spread of COVID-19, such as lockdowns, home isolation, and social distancing. Reports suggested that stressful events such as natural disasters and man-made traumas exerted significant mental health impacts and could result in conditions such as posttraumatic stress disorder (PTSD) and depression (Kopala-Sibley et al., 2016; Plexousakis et al., 2019; Schwartz et al., 2019). However, public health authorities and healthcare researchers had paid more attention to the biological and physical effects rather than to the mental health implications of COVID-19.

Post-traumatic stress disorder is a chronic impairment disorder that often occurs after exposure to severe stressors, like combat, nature disaster, or other events (White et al., 2015). It is characterized by re-experience and avoidance symptoms as well as negative alterations in cognition and arousal (Miao et al., 2018). PTSD was first noticed by public during and after the military of the United States in Afghanistan and Iraq, and then a large number of relevant studies occurred in this field. However, underlying mechanisms of PTSD and specific treatment for it remain unclear. Therefore, due to the significant medical, social and even financial problems, PTSD is remarkable both for individuals and society, people suffering from PTSD or under traumatic exposure need to know about the risk and effects of PTSD.

Previous studies based on the SARS outbreak indicated that mental health problems were common among SARS survivors and authors referred to SARS as a “mental health catastrophe” (Maunder, 2009). Mak et al. (2009) reported that among 90 residents of Hong Kong who were infected with SARS and survived, 25.6% had PTSD and 15.6% had depressive disorders 30 months after infection. Another study by Leung et al. showed the trend of the population’s anxiety followed the trend of the number of daily new cases (Leung et al., 2005). One prospective cohort study on college students indicated that the prevalence of anxiety increased markedly in the early phase of the SARS but gradually reduced over time (Cheng and Cheung, 2005). And for now, the COVID-19 pandemic has brought into focus the mental health of affected populations, such as patients, medical staff, children, and older adults (Fu et al., 2021). Mental health of college students during COVID-19 pandemic were gradually of particular interest, as they experienced nationwide school closures, sudden change in students’ learning environment, loss of internships, on-campus jobs, and other opportunities (J. Lee et al., 2021). Chi et.al conducted a study among college students in mainland China during the beginning of COVID-19 and indicated that 30.8% (95% CI: 28.8–32.8%) of the participants presented relevant PTSD symptoms (Chi et al., 2020). Another survey conducted in the US found that among 2031 participants, 48.14% showed a moderate-to-severe level of depression, 38.48% showed a moderate-to-severe level of anxiety, and 18.04% had suicidal thoughts, the majority of participants (71.26%) indicated that their stress/anxiety levels had increased during the COVID-19 pandemic (Wang et al., 2020).

A growing number of researchers investigated the prevalence of PTSD among college students, and the reported prevalence ranged from 2.7 to 66.7% (Tang et al., 2020; Abdel-Aziz et al., 2022). But the overall prevalence of college students worldwide was deficient for raising awareness and providing appropriate interventions. In addition, the findings of these researches converged on the uptick of mental health issues among college students, but the contributing factors might not be generalizable to populations worldwide. As we observed the prevalence of PTSD varied among college students from different countries, and the most significant difference existed between students in Egypt (Africa, 66.7%) and China (Asia,2.7%; Tang et al., 2020; Abdel-Aziz et al., 2022). Thus, we inferred that factor like continents and national income participated in the different outcome of studies. In addition, previous studies had reported several mental health problems were high prevalent among medical students (Pacheco et al., 2017). Therefore, there was an urgent need to comprehensively assess the effects of the COVID-19 pandemic on mental health especially the prevalence of PTSD among college students.

Collectively, the present study aimed to provide a more accurate representation of PTSD in college students during the COVID-19 pandemic by performing meta-analyses by continents, national income, and study majors and comparing the results with estimated pooled prevalence, to assist policymakers to develop relative policies and help clinical practitioners to provide services to the affected populations in time.

## 2. Materials and methods

The present study was conducted following the Preferred Reporting Item for Systematic Reviews and Meta-analyses (PRISMA) statement. And the study protocol was registered in the PROSPERO (CRD42022382828) before data extraction.

### 2.1. Search strategy

Electronic databases including PubMed, Embase, and Web of Science databases were used for systematically searching for related literature that was published in English from March 2020 to September 2022. The keywords used for search strategies were as follows: “COVID-19” OR “SARS-CoV-2” AND “college students” or “university students” or “undergraduate students” or “bachelor students” or “graduate students” or “doctorate students” or “higher education students” AND “post-traumatic stress disorder” OR “PTSD.” Combinations of the above keywords were modified to optimize the search results in the databases. All of the included literature were screened manually to identify potential articles.

### 2.2. Inclusion and exclusion criteria

The EndNote X7 program (Clarivate Analytics, Philadelphia) was used to import and document articles, and duplications were first excluded. The preliminary screening was conducted by reading the titles and abstracts of the articles. After that, the articles were further screened by two independent reviewers (BH and XY) according to the inclusion and exclusion criteria. A third researcher (XT) was consulted to assist in the judgment in case of disagreement.

The inclusion criteria were as follows: (a) studies related to COVID-19; (b) studies published in English; (c) the study design included cross-sectional studies; (d) the study population consisted of college or university students; (e) the presence of PTSD was measured with clinical interviews or questionnaires according to DSM diagnostic criteria; and (f) studies containing a clear description of the prevalence of PTSD. The exclusion criteria were as follows: (a) the college or university students with mental illness already; (b) articles that were inconsistent with the study aim, re-published, books, editorials, conference abstracts, letters to the editors, viewpoints, case presentations, and brief communications; and (c) lacking sufficient data required to conduct the basic analysis.

### 2.3. Data extraction

The extracted data were as follows: (1) the first author of the study; (2) publication year; (3) the country surveyed; (4) study design; (5) sample size; (6) the assessment tools; (7) prevalence and standard error (SE); (8) relevant subgroup data. SE could be calculated using the formula SE=$\sqrt{P}\times \left(1-P\right)÷N$ when SE value was not presented in the article, and *P* was the proportions of cases reported in the article, *N* was the sample size of the prevalence estimate. For a longitudinal study in our meta-analysis, we chose the prevalence surveyed during the COVID-19 pandemic.

### 2.4. Data analysis

Review Manager 5.4 (RevMan 5.4, The Cochrane Collaboration, London) was used to conduct the meta-analytic calculations. The pooled prevalence of PTSD, 95% confidence intervals (CI), *Z*-value, and heterogeneity test values were calculated, respectively. Heterogeneity was estimated by Cochran’s Q-test (Chi-square) and *I*^2^ statistics, a *value of p* of Chi-square < 0.10 indicated heterogeneity of the estimates of the prevalence rates, and *I*^2^ of 25, 50 and 75% indicated low, moderate and high heterogeneity, respectively. The random effect mode was used to account for expected heterogeneity between studies. For the source of heterogeneity, we conducted the subgroup analysis divided by the economic level of countries, majors of college students, and the continents. Finally, we used funnel plots to explore whether publication bias existed.

### 2.5. Quality assessment

The Joanna Briggs Inventory (JBI) Checklist was used to evaluate study quality by two researchers (BH and XY) independently. The quality was evaluated based on the following items: (a) sample frame; (b) sampling; (c) sample size; (d) description of subjects and setting; (e) sample coverage of the data analysis; (f) validity of the methods to identify the condition; (g) standardization and reliability of the methods to measure the con by the edition of all participants; (h) statistical analysis; and (i) response rate (Table 1). An item was scored ‘0’ when it was rated ‘no’, ‘unclear’, or ‘not applicable’, and was scored ‘1’ when it was rated ‘yes’. The overall scores ranged from 0 to 9, and the quality of the included eligible studies was assessed as follows: 0–3, 4–6, and 7–9 indicated a high, moderate, and low risk of bias, respectively. And when disagreements occurred in the assessments, they were resolved by another author (XT).

**Table 1:** Risk of bias assessment for included studies.

Author (year)	a	b	c	d	e	f	g	h	i	Scores
Abdel-Aziz et al. (2022)	*	*	/	*	*	*	*	*	*	8
Cam et al. (2022)	*	*	*	*	*	*	*	*	*	9
Camilleri et al. (2022)	*	*	*	*	*	*	*	*	*	9
Chi et al. (2020)	*	*	*	*	*	*	*	*	*	9
Dixit et al. (2022)	*	*	/	*	*	*	*	*	*	8
El Maouch et al. (2022)	*	*	*	*	*	*	*	*	*	9
Elbarazi (2022)	*	*	*	*	*	*	*	*	NA	8
Feng et al. (2022)	*	*	*	*	*	*	*	*	*	9
Fila-Witecka et al. (2022)	*	*	*	*	*	*	*	*	*	9
Gao et al. (2021)	*	*	*	*	*	*	*	*	*	9
Giusti et al. (2020)	*	*	/	*	*	*	*	*	*	8
Ifthikar et al. (2021)	*	*	/	*	*	*	*	*	*	8
Jardon and Choi (2022)	*	*	*	*	*	*	*	*	/	8
Joseph et al. (2022)	*	*	*	*	*	*	*	*	/	8
Kim et al. (2022)	*	*	*	*	*	*	*	*	/	8
Kim et al. (2022)	*	*	*	*	*	*	*	*	*	9
Kumar et al. (2020)	*	*	*	*	*	*	*	*	*	9
Kumar et al. (2021)	*	*	*	*	*	*	*	*	*	9
C. M. Lee et al. (2021)	*	*	*	*	*	*	*	*	/	8
Lee et al. (2022)	*	*	*	*	*	*	*	*	*	9
Li et al. (2021)	*	*	*	*	*	*	*	*	*	9
Li et al. (2021)	*	*	*	*	*	*	*	*	*	9
Lin et al. (2021)	*	*	*	*	*	*	*	*	*	9
López-Castro et al. (2021)	*	*	*	*	*	*	*	*	*	9
Ochnik et al. (2021)	*	*	*	*	*	*	*	*	*	9
Rogowska et al. (2022)	*	*	*	*	*	*	*	*	*	9
Saali et al. (2022)	*	*	*	*	*	*	*	*	*	9
Wang et al. (2022)	*	*	*	*	*	*	*	*	NA	8
Soltan et al. (2021)	*	*	*	*	*	*	*	*	NA	8
Bijia et al. (2020)	*	*	*	*	*	*	*	*	*	9
Sultana et al. (2021)	*	*	*	*	*	*	*	*	*	9
Sun et al. (2021)	*	*	*	*	*	*	*	*	NA	8
Tang et al. (2020)	*	*	*	*	*	*	*	*	*	9
Udgiri et al. (2021)	*	*	*	*	*	*	*	*	NA	8
Wathelet et al. (2021)	*	*	*	*	*	*	*	*	NA	8
Xie et al. (2021)	*	*	*	*	*	*	*	*	*	9
Xu et al. (2021)	*	*	*	*	*	*	*	*	*	9
Zhang et al. (2021)	*	*	*	*	*	*	*	*	*	9

“*” means that item was scored ‘1’ because it was rated ‘yes’.

## 3. Results

### 3.1. Search results

**Figure 1:**
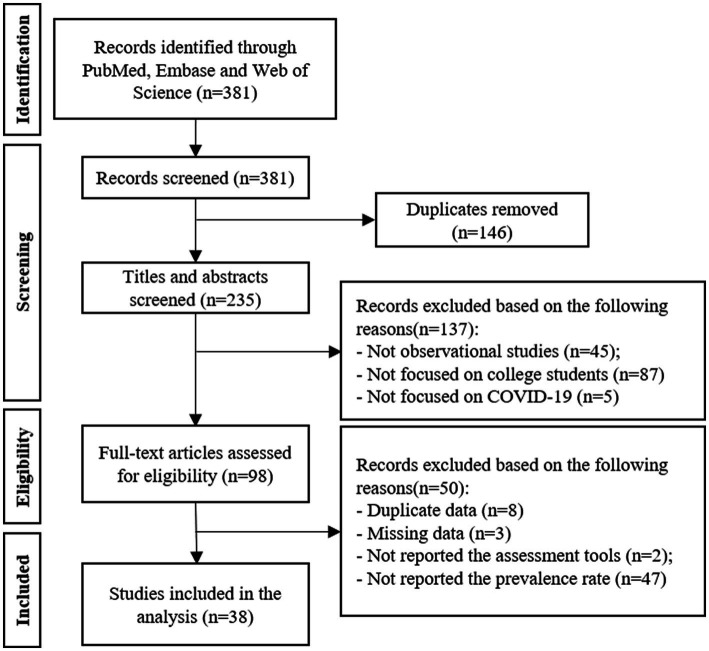
PRISMA flow diagram of the literature search strategy. PRISMA, Preferred Reporting Items for Systematic Reviews and Meta-analyses. PTSD, post-traumatic stress disorders.

In total, 381 articles were retrieved from three electronic databases and then imported into the Endnote X7. After the removal of 146 duplications, 235 articles were next screened by two reviewers. By reading titles and abstracts, 137 articles were eliminated since they did not meet the inclusion criteria listed previously. Then 98 potential original articles were further screened by reading the full context and 60 articles were excluded because of the lack of detailed data mentioned in the inclusion criteria. Finally, 38 articles were included in the present meta-analysis (Figure 1).

### 3.2. Study characteristics

The descriptive characteristics of the 38 included studies were presented in Table 2. Totally 95,375 college students were included in the present meta-analysis, and all of the included studies were cross-sectional studies. Included studies from 16 countries, that belonged to four continents (See Table 2). We extracted the information about mean age and standard deviation from all of the included studies if possible, and college students surveyed were at a similar age. For the assessment of PTSD, 14 studies used IES-R (Impact of Event Scale-Revised), six studies used PCL-C (Posttraumatic Stress Disorder Checklist—Civilian Version), four studies used PCL-5 (PTSD Checklist for the DSM-5), two studies used PCL (The Abbreviated PTSD Checklist), PC-PTSD (the Primary Care PTSD Screen), IES-6 (6-item Impact of Event Scale-Revised), PCL-S (17-item PTSD check list-specific version), and IES (Impact of Event scale) separately to assess PTSD. In addition, one study used GPS-PTSS (Global Psycho-trauma Screen), The COVID Stress Scale, PC-PTSD-5 (Primary Care PTSD Screen DSM-5), IES-R-K (Impact of Event Scale-Revised), respectively, to assess PTSD (See Table 2).

**Table 2:** Descriptive characteristics of studies (Table view).

Author (year) Ref No.	Study continent	Income level	Sample size (N)	Number of PTSD cases (prevalence rate)	Majors of students	Assessment tools	Research time
Abdel-Aziz et al. (2022)	Africa	Lower-middle	81	54 (66.7%)	Medical	GPS-PTSS	July–September 2020
Cam et al. (2022)	Asia	Upper-middle	1,095	378 (34.5%)	Non-medical	IES-R	May 11–15 2020
Camilleri et al. (2022)	North America	High	608	93 (15.3%)	Non-medical	IES-R	August–September 2020
Chi et al. (2020)	Asia	Upper-middle	2038	628 (30.8%)	Non-medical	PCL	February 12–17 2020
Dixit et al. (2022)	Asia	High	24	7 (29.2%)	Medical	IES-R	December 2020
El Maouch et al. (2022)	Asia	Upper-middle	852	203 (23.8%)	Non-medical	IES-R	November 2021–March 2022
Elbarazi (2022)	Africa	Lower-middle	1,195	273 (22.8)	Non-medical	PCL	December 2021–February 2022
Feng et al. (2022)	Asia	Upper-middle	2070	147 (7.1%)	Non-medical	PCL-C	December 2020–January 2021
Fila-Witecka et al. (2022)	Europe	High	1,053	689 (65.4%)	Non-medical	IES-R	May–June 2020
Gao et al. (2021)	Asia	Upper-middle	1,532	46 (3.0%)	Medical	IES-R	April 12–23 2020
Giusti et al. (2020)	Europe	High	103	14 (13.6%)	Non-medical	IES-R	March 16–May 4 2020
Ifthikar et al. (2021)	Asia	High	309	114 (36.9%)	Medical	IES-R	June 16–August 18 2020
Jardon and Choi (2022)	North America	High	174	57 (32.8%)	Medical	The COVID Stress Scales	March–Apr 2021
Joseph et al. (2022)	North America	High	207	56 (27.1%)	Medical	IES-R	May–September 2020
Kim et al. (2022)	North America	High	4,524	1,306 (28.9%)	Non-medical	PC-PTSD	March 2–May 9 2020
Kim et al. (2022)	Asia	High	400	129 (32.3%)	Non-medical	IES-6	June–July 2021
Kumar et al. (2020)	Asia	Lower-middle	331	75 (22.7%)	Medical	IES-R	April 4–16 2020
Kumar et al. (2021)	Asia	Lower-middle	420	161 (38.3%)	Medical	IES-R	July–December 2020
C. M. Lee et al. (2021)	North America	High	741	188 (25.4%)	Medical	PC-PTSD-5	April–May 2020
Lee et al. (2022)	Asia	High	270	28 (10.4%)	Medical	IES-R-K	2021
Li et al. (2021)	Asia	Upper-middle	6,348	950 (15.0%)	Medical	PCL-C	March 8–24 2020
Li et al. (2021)	Asia	Upper-middle	4,355	708 (16.3%)	Non-medical	IES-R	April 26–29 2020
Lin et al. (2021)	Asia	Upper-middle	1,022	163 (15.9%)	Non-medical	PCL-C	June 27–30 2021
López-Castro et al. (2021)	North America	High	909	43 (4.7%)	Non-medical	PC-PTSD-5	May 1–31 2020
Ochnik et al. (2021)	Not available	Not available	1741	570 (32.7%)	Non-medical	PCL-S	October–December 2020
Rogowska et al. (2022)	Not available	Not available	3,230	741 (22.9%)	Non-medical	PCL-S	November 2020
Saali et al. (2022)	North America	High	108	8 (7.4%)	Medical	PCL-5	June–July 2020
Wang et al. (2022)	Asia	Upper-middle	3,641	1,245 (34.2%)	Non-medical	IES-6	February 23–May 5 2020
Soltan et al. (2021)	Africa	Lower-middle	282	153(54.3%)	Medical	IES-R	May 1–June 2020
Bijia et al. (2020)	North America	High	261	98 (37.5%)	Non-medical	PCL-C	Not available
Sultana et al. (2021)	Asia	Lower-middle	3,997	1,635 (40.9%)	Non-medical	IES	May 29–July 22 2020
Sun et al. (2021)	Asia	Upper-middle	1912	338 (17.7%)	Non-medical	IES	March 20–April 10 2020
Tang et al. (2020)	Asia	Upper-middle	2,485	67 (2.7%)	Non-medical	PCL-C	February 20–27 2020
Udgiri et al. (2021)	Asia	Lower-middle	80	25 (31.3%)	Medical	IES-R	Not available
Wathelet et al. (2021)	Europe	High	22,883	4,456 (19.5)	Non-medical	PCL-5	June 15–July 15 2020
Xie et al. (2021)	Asia	Upper-middle	8,879	615 (6.9%)	Non-medical	PCL-5	April 20–26 2020
Xu et al. (2021)	Asia	Upper-middle	11,254	952 (8.5%)	Non-medical	PCL-5	June 29–July 18 2020
Zhang et al. (2021)	Asia	Upper-middle	3,961	870 (22.0%)	Non-medical	PCL-C	April 4–24 2020

Global Psycho-trauma Screen (GPS-PTSS); Impact of Event Scale-Revised (IES-R); The Abbreviated PTSD Checklist (PCL); Posttraumatic Stress Disorder Checklist–Civilian Version (PCL-C); the Primary Care PTSD Screen (PC-PTSD); Impact of Event Scale-6 (IES-6); Primary Care PTSD Screen DSM-5 (PC-PTSD-5); Impact of Event Scale-Revised (IES-R-K); 17-item PTSD checklist—specific version (PCL-S); PTSD Checklist for the DSM-5(PCL-5); 6-item Impact of Event Scale-Revised (IES-6); Impact of Event scale (IES).

### 3.3. The pooled prevalence of PTSD

**Figure 2:**
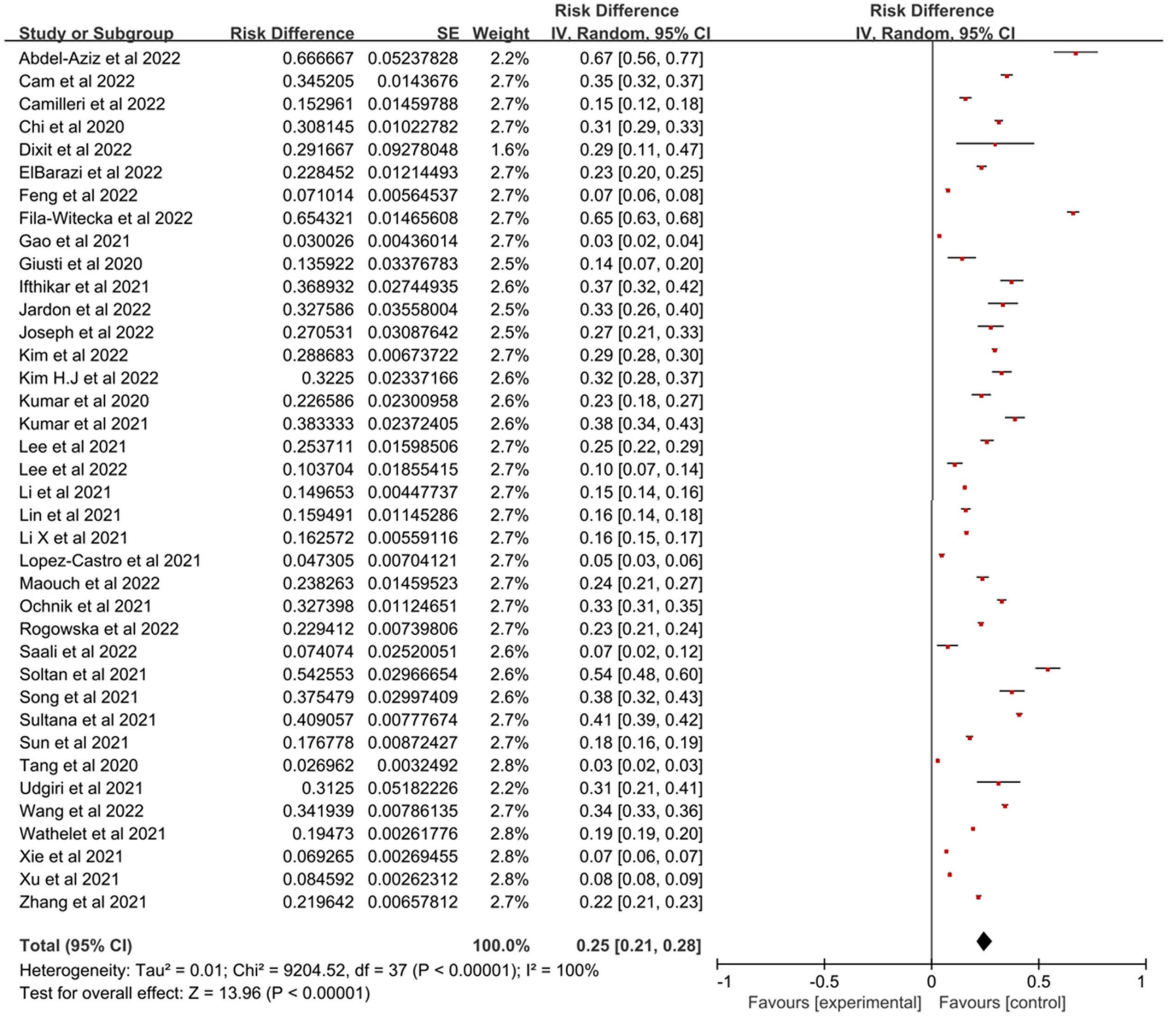
Forest plot showed the prevalence of PTSD among college students during the COVID-19 pandemic. CI, confidence intervals.

Figure 2 showed the forest plot for the complete dataset. Across the 38 studies with 95,375 college students, the pooled prevalence for all the students was 25% (*N* = 38, *n* = 95,375, 95% confidence interval: 21–28%) with high heterogeneity (*I*^2^ = 99.9%, *p* < 0.001).

### 3.4. Subgroup analysis

**Figure 3:**
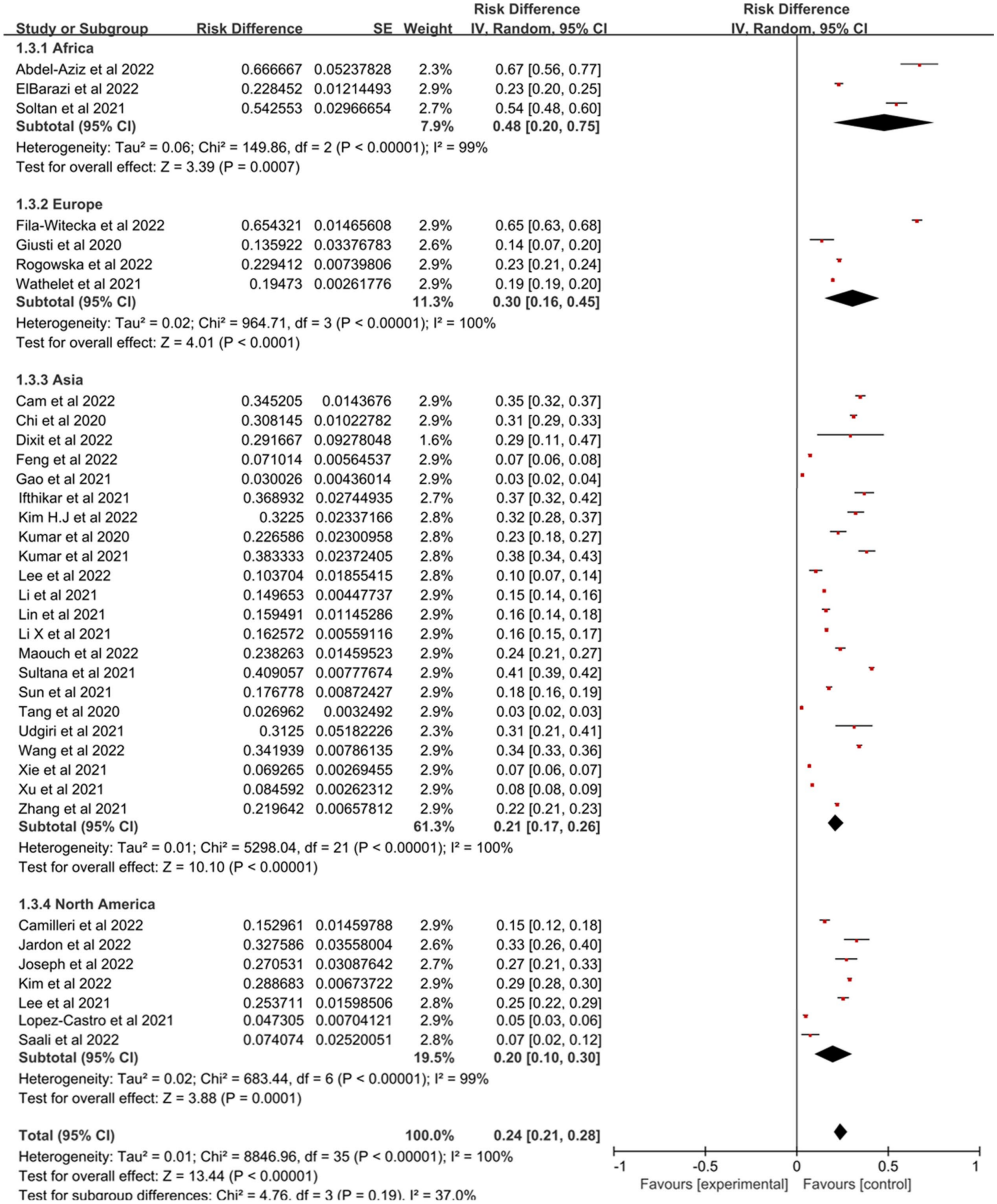
Subgroup analysis based on different continents for estimating PTSD.

**Figure 4:**
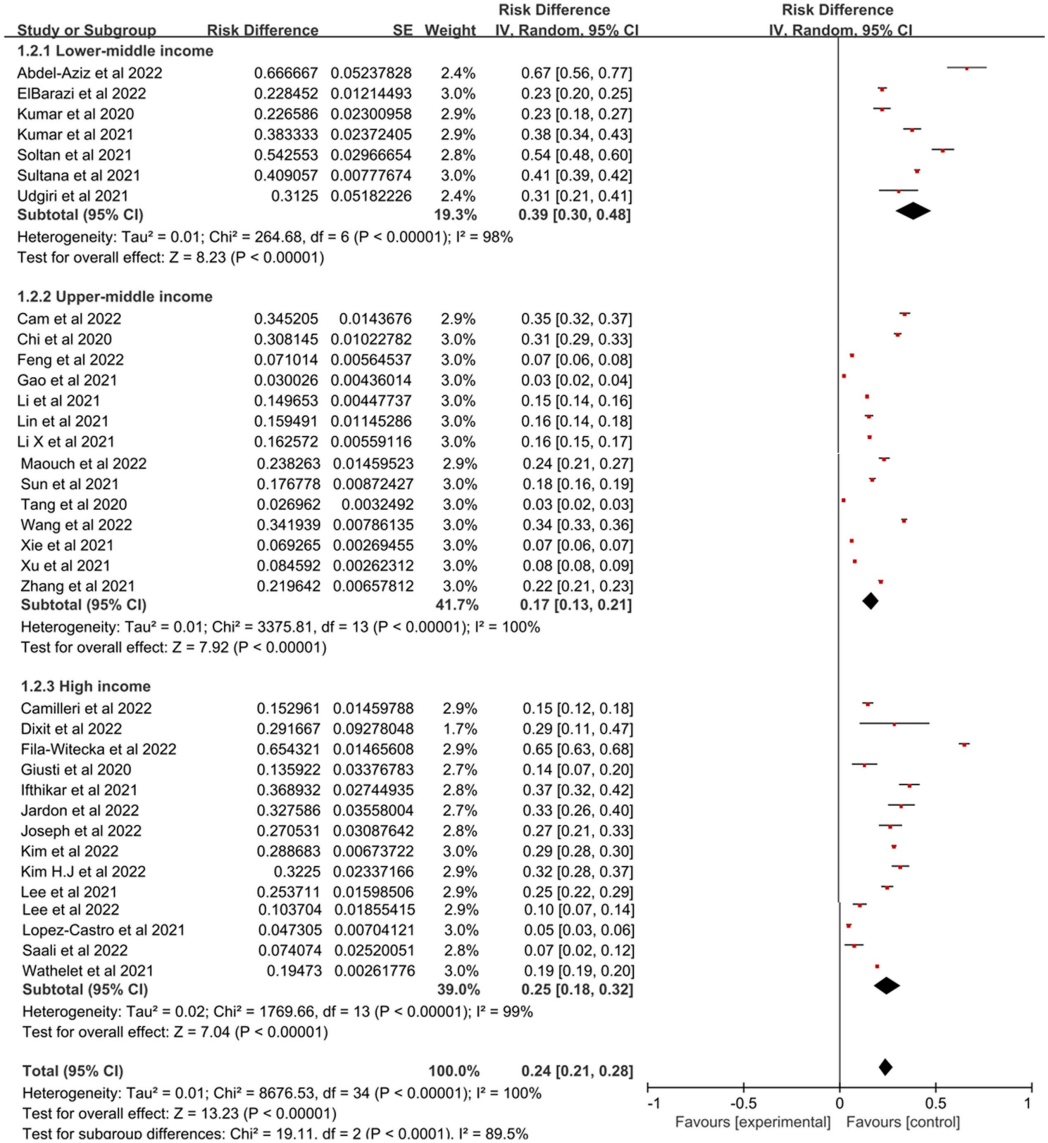
Subgroup analysis based on different national income levels for estimating PTSD.

**Figure 5:**
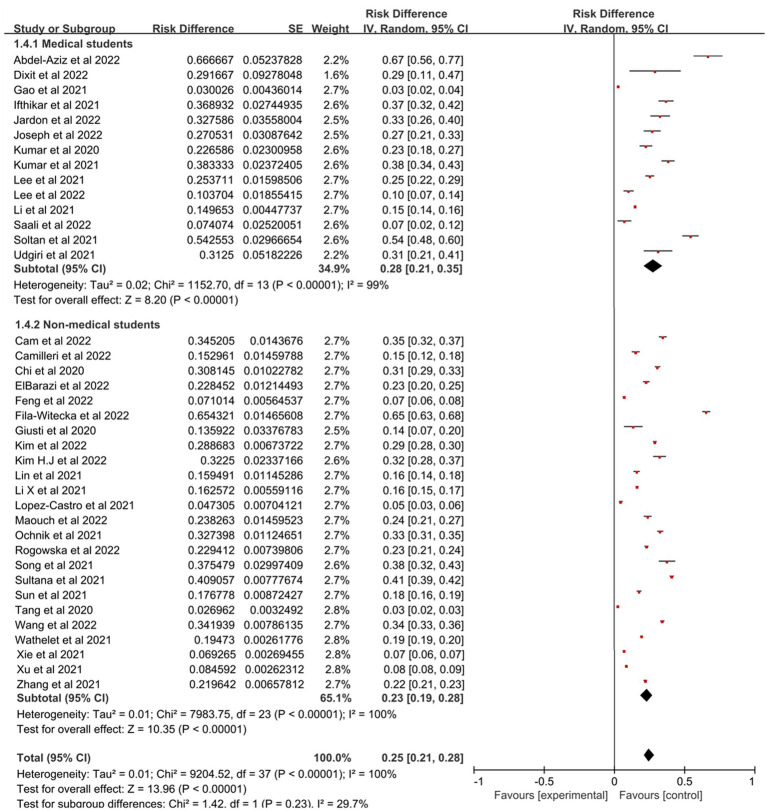
Subgroup analysis based on different study majors for estimating PTSD.

Prevalence estimates of PTSD among college students were statistically significant (*p* < 0.00001) when stratified by geographical regions (Figure 3), with Africa having the highest prevalence (48, 95% CI: 20–75%), followed by Europe (30, 95% CI: 16–45%), Asia (21, 95% CI: 17–26%) and North America which was 20% (95% CI: 10–30%). Figure 4 represented the prevalence estimates of PTSD among college students were statistically significant (*p* < 0.00001) that were stratified by national income level, with lower-middle-income level having the highest prevalence (39, 95% CI:30–48%), followed by high-income level (25, 95% CI:18–32%) and upper-middle-income level which was 17% (95% CI:13–21%). Finally, in terms of study majors of college students, the prevalence estimates of PTSD among medical college students (28, 95% CI:21–35%) was higher than that of non-medical college students (23, 95% CI,19–28%; Figure 5). Collectively, in comparison with the pooled prevalence of PTSD (25%), subgroups of Africa and Europe, lower-middle-income countries and medical college students possessed higher prevalence estimates.

### 3.5. Quality assessment and publication bias

Assessment for the risk of bias ascertained for each article was listed in Table 1. Overall, the overall score ranged from 8 to 9 which indicated a low risk of bias. Publication bias was not observed among the analyses where it was possible to systematically assess publication bias using funnel plots (Supplementary Figure S1).

## 4. Discussion

To our knowledge, the present study is the first meta-analysis to investigate the prevalence of PTSD symptoms among college students worldwide during the COVID-19 pandemic. The pooled prevalence of PTSD during the COVID-19 pandemic is 25% (95%CI: 21–28%) in this study, compared with the prevalence in the general population ranging from 1 to 10%, and in college students ranging from 6 to 17% before the COVID-19 pandemic (Read et al., 2011). Higher prevalence can be explained by the decrease in face-to-face social interaction, long-time online learning, fear associated with the pandemic, and fewer opportunities to exercise for students during the COVID-19 pandemic (Feng et al., 2014; Nicola et al., 2020; Rogers et al., 2020). In all, the present study can be a great warning about the mental health of college students during the COVID-19 pandemic. Mental health care should not be, therefore, underestimated at present, especially for college students.

In this study, we observed that the prevalence of PTSD among college students on different continents varies from each other. The prevalence of PTSD among African college students is the highest (48%), while the prevalence among North American college students is the lowest (20%). With the rapidly increasing number of newly confirmed cases, three major factors are impacting the population and might lead to mental health problems: firstly, the direct impact of the disease, particularly near-death experiences during illness, and isolation from loved ones; secondly, restriction limits the social support and supply of food and medication; thirdly, uncertainty and stress resulting from loss of jobs and livelihoods (Semo and Frissa, 2020). Previous studies have reported significant differences in risk of acquiring COVID-19 between the continents, in which Asia and North America showed lower risks, and that lead to less direct or indirect impact of COVID-19 in North America and Asia (Sunjaya et al., 2022). Different social cultures, lifestyles, and political values in different continents might lead to the discrepant prevalence of PTSD among college students across continents. In addition, poor recognition of other outbreak-related epidemiologic terms might lead to passive attitudes to intervention strategies and measures (Zhang and Ba-Thein, 2022). It is also known that North America and Europe possess better medical condition than African, which partially interpret the difference of prevalence of PTSD among continents. Moreover, population density varies across continents, which also caused heterogeneity in the outcome. As Shuwiekh et al. (2022) have reported more densely populated countries like Egypt had a greater mental health impact than the countries with lower population density like Algeria and Saudi Arabia.

Factors like economic and income level also affect the spread of COVID-19 and its subsequent mental health impact like PTSD. Subgroup analyses based on the income levels (lower-middle income/upper-middle income/high-income) among countries indicate that the income levels of countries correlate with the prevalence of PTSD among college students. Lower-middle-income countries possess the highest prevalence since the reduction in household income is significantly associated with an increased risk of incident mental disorders (Sareen et al., 2011; Pieh et al., 2020). For example, it has been shown that lower socioeconomic status is linked to increased levels of PTSD and depression among people who have been exposed to trauma (Ochnik et al., 2021). Similarly, material assets are significantly related to mental health outcome, as the economic crises brought about by COVID-19 have markedly increased vulnerabilities to psychiatric symptomatology (Rudenstine et al., 2022). Moreover, the lower economic income can cause additional frustration due to incapable to cover the adequate need for supplies, medical attention and maintaining previous lifestyles, as indicated in previous studies on the consequences of quarantine (Alejandro-Salinas et al., 2022). A study based on family factors also has verified the relationship between the prevalence of PTSD and family background, which has shown university students from extremely poor families had the highest prevalence of PTSD, in contrast to the lowest level among students from wealthy families (Zhang et al., 2021). During the COVID-19 pandemic, some college students in developing countries like Pakistan, are unable to afford laptops because of electricity shortages and connectivity issues and may have apprehensions about their practical capabilities as a professional as they are unable to learn in hospitals through clinical rotations and internships (Gonzalo et al., 2009; Arima et al., 2020; Gallagher and Schleyer, 2020).

Finally, subgroup analysis that based on medical and non-medical students has shown, the prevalence of PTSD among medical college students during the COVID-19 pandemic is significantly higher than among non-medical college students. Related reasons are inferred as follows: first of all, medical students need to complete some on-site operation and internship courses in hospitals, but hospitals are key areas for epidemic control, which has caused more pressure on medical students. Then, in the process of fighting against COVID-19, the medical staff’s hard work and lack of corresponding supply guarantee in the early stage have made them go through a difficult period, which may have an impact on the medical students’ practice choice (Wang et al., 2022). Finally, during the period of isolation, separation from loved ones, loss of freedom, boredom and uncertainty about disease conditions may also bring psychological distress and disorder symptoms to medical staff.

The COVID-19 pandemic is one of the risk factors leading to college students’ psychological problems, which should be solved as an urgent matter of public health. Isolation and treatment of infected persons and possible expansion of the vaccination population are effective measures to control the COVID-19 pandemic (Frederiksen et al., 2020). Given that medical college students are more likely to suffer mental problems, we should give more attention to medical students and provide psychological support and material services to reduce their risk of developing PTSD and other mental problems. And for those who have suffered from PTSD, online and telephone support can be provided for help, since that has already been verified as an effective emergency measure in many countries. Popularizing medical knowledge about COVID-19 can also help reduce panic and the risk of mental problems among college students (Wang et al., 2020).

## 5. Conclusion

The objective of the meta-analysis was to assess the prevalence of PTSD in college students worldwide by continent and national income levels during the COVID-19 pandemic. The findings of our meta-analysis suggested that 25% of college students experienced PTSD during COVID-19, and the prevalence varied among different continents, different national income levels, and different study majors of college students. The highlighting of the high prevalence of college students’ PTSD, and comprehensive analysis of potential risk factors could help to raise caution among college students during COVID-19. To better understand the prevalence of PTSD among college students worldwide, future studies can use standard and reliable questionnaires to conduct surveys reasonably sampled from the world. Meanwhile, we should take measures to reduce the impact of the pandemic on people’s mental health urgently.

## 6. Limitation of the study and future direction

Limitations of our study must be noted to provide a better interpretation of the present study. Firstly, all of the included studies were cross-sectional studies, which might not provide sufficient evidence for the association between the COVID-19 pandemic and the increased prevalence of PTSD among college students. In addition, the heterogeneity of the studies included in our meta-analysis was relatively high, and heterogeneities of subgroup analysis based on the continents, national income and study major were also relatively high. However, the present study included 95,375 college students, which was quite a large sample for meta-analysis to assess the pooled prevalence of PTSD among college students worldwide, and might not affect the overall prevalence of PTSD among college students.

The results of the study highlighted the need for future research to pay attention to mental health of vulnerable population (such as patients, children, pregnant women and college students) after exposure to emergent events of public health. Additionally, further study is needed to determine the effects of the COVID-19 on college students’ mental health and well-being in its later phases rather than the peak period. Based on relative studies during the SARS outbreak, the effects of the COVID-19 on students might linger for a time beyond the peak of the COVID-19 itself (McAlonan et al., 2007).

## Data availability statement

The original contributions presented in the study are included in the article/Supplementary material, further inquiries can be directed to the corresponding author.

## Author contributions

XT designed the methodology of the study, supervised and performed article screening, data extraction, statistical analyses, critically reviewed and revised the manuscript, and accepted responsibility for the integrity of the data analyzed. XY and BH performed article screening, data extraction, and statistical analyses and drafted the manuscript. All authors had read and approved the final version of the manuscript to be submitted.

## Conflict of interest

The authors declare that the research was conducted in the absence of any commercial or financial relationships that could be construed as a potential conflict of interest.

## Publisher’s note

All claims expressed in this article are solely those of the authors and do not necessarily represent those of their affiliated organizations, or those of the publisher, the editors and the reviewers. Any product that may be evaluated in this article, or claim that may be made by its manufacturer, is not guaranteed or endorsed by the publisher.
