# Supplementary material for: The prevalence of post-traumatic stress disorder in college students by continents and national income during the COVID-19 pandemic: a meta-analysis
Source: Front Psychol. 2023 May 12;14:1129782. doi: 10.3389/fpsyg.2023.1129782 (PMC10217783; doi:10.3389/fpsyg.2023.1129782)
Supplement: Supplementary file 1 [file Table_1.DOCX]

Supplementary Material

The prevalence of post-traumatic stress disorder in college students by continents and national income during the COVID-19 pandemic: a meta-analysis

Biao Hu, Xiling Yang, Xiaoqian Tuo^*^

*** Correspondence:** Corresponding Author: island19940331@stu.xjtu.edu.cn

# Supplementary Figure


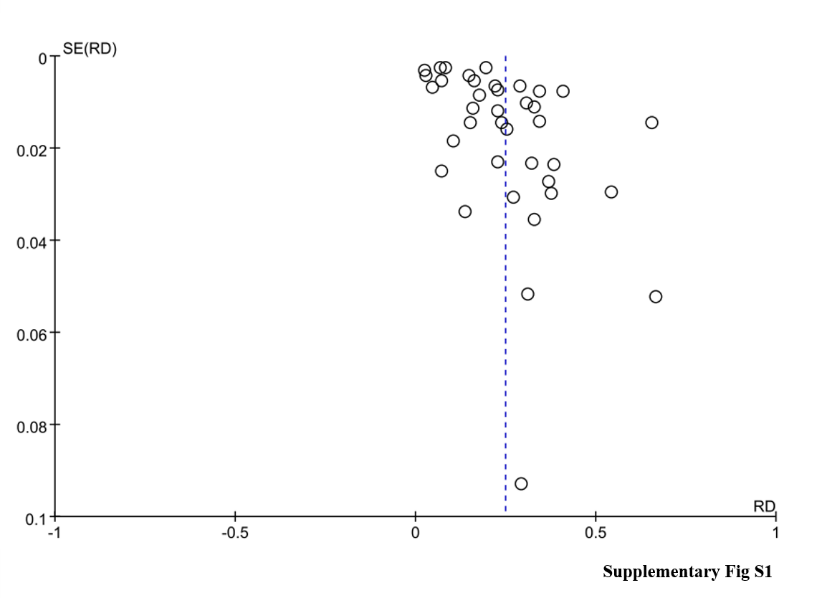


**Supplementary Figure 1.** Funnel plot of included studies in the analysis to assess publication bias.
